# Understanding the heterogeneity of anxiety using a translational neuroscience approach

**DOI:** 10.3758/s13415-024-01162-3

**Published:** 2024-02-14

**Authors:** Carly M. Drzewiecki, Andrew S. Fox

**Affiliations:** 1 California National Primate Research Center, University of California, Davis, CA USA; 2 Department of Psychology, University of California, Davis, CA USA

**Keywords:** Anxiety disorders, Computational modeling, Animal models, Amygdala, Fear and anxiety

## Abstract

Anxiety disorders affect millions of people worldwide and present a challenge in neuroscience research because of their substantial heterogeneity in clinical presentation. While a great deal of progress has been made in understanding the neurobiology of fear and anxiety, these insights have not led to effective treatments. Understanding the relationship between phenotypic heterogeneity and the underlying biology is a critical first step in solving this problem. We show translation, reverse translation, and computational modeling can contribute to a refined, cross-species understanding of fear and anxiety as well as anxiety disorders. More specifically, we outline how animal models can be leveraged to develop testable hypotheses in humans by using targeted, cross-species approaches and ethologically informed behavioral paradigms. We discuss reverse translational approaches that can guide and prioritize animal research in nontraditional research species. Finally, we advocate for the use of computational models to harmonize cross-species and cross-methodology research into anxiety. Together, this translational neuroscience approach will help to bridge the widening gap between how we currently conceptualize and diagnose anxiety disorders, as well as aid in the discovery of better treatments for these conditions.

## Introduction

Anxiety disorders are characterized by debilitating, extreme, and chronic experiences of fear and anxiety. They are among the most prevalent psychiatric disorders; estimates suggest that more than one in four people will experience an anxiety disorder in their lifetime (Bandelow & Michaelis, 2015; Kessler et al., 2012). Anxiety disorders often are comorbid with other disorders, including depression, substance abuse, eating disorders, and premenstrual dysphoric disorders (Merikangas & Swanson, 2009; Swendsen et al., 2010; Yen et al., 2020). In short, these disorders are one of the largest contributors to days lost to disability and impose an extreme burden on public health (Rice & Miller, 1998; Yang et al., 2021b).

Despite extensive research in this area, existing cognitive, behavioral, and/or pharmacological treatments for anxiety disorders remain suboptimal. Although many patients respond to existing treatments, responses often are incomplete, failing to fully ameliorate symptoms, even when treatments are combined (Carpenter et al., 2018; Slee et al., 2019; Szuhany & Simon, 2022). Between one-third to one-half of patients do not respond to front-line treatments, and often less than half of patients ever fully achieve remission (Bandelow et al., 2014; Bereza et al., 2012; Pollack et al., 2008). These suboptimal outcomes underscore the need for basic science to produce a refined understanding of the biological mechanisms that give rise to anxiety disorders and motivate new treatments.

Anxiety disorders are characterized by the subjective experience of fear and anxiety, but there is substantial heterogeneity that persists over time and across contexts in the outward presentation of anxiety disorders. Basic science in preclinical animal models is critical for uncovering causal biological factors but often is limited by a focus on a restricted set of behaviors across a limited number of contexts. Here, we discuss the heterogeneity of anxiety disorders, how phenotypic heterogeneity relates to our current understanding of fear- and anxiety-related neurocircuitry in animal models, and highlight emerging approaches that can help bridge the gap between basic and clinical science. We argue that a refined understanding of the neurobiology of anxiety disorders necessitates a translational neuroscience approach that incorporates a broader set of assays and the utilization of computational modeling. A more complete understanding of these disorders will be a crucial step toward the development of effective treatments to alleviate suffering in patients.

## Defining fear and anxiety

The central, defining feature of all anxiety disorders is the extreme experience of anxiety. Unfortunately, there is little evidence that people’s use of the word “anxiety” is consistent or uniquely associated with a specific biological state. In fact, many people use other words, such as “fear,” “afraid,” or “worry” when describing their anxieties. Optimally diagnosing and treating anxiety disorders implicitly relies on a shared understanding of emotion, requiring clinicians and patients to use a consistent definition of these words. However, the lack of objective definitions for the terms “fear” and “anxiety” persist (Shackman & Fox, 2016). Models that redefine these common lexical terms have been proposed, with “fear” as a response to acute and phasic threats, and “anxiety” as a response to sustained and uncertain threats. (Davis et al., 2010). However, the evidence that these different emotions are dissociable at the level of phenomenology, physiology, behavior, and brain remains unclear (Shackman & Fox, 2016).

The potential mismatch between the definitions of anxiety-relevant words across patients, clinicians, and scientists represents a major problem for understanding the heterogeneity of disorder. If scientists are using the same words in different ways, this can provide a major barrier to the translation of their findings. For example, the use of the phrase “fear-conditioning” for the study of tone-shock learning in animals implies that these findings are directly relevant to the understanding of the human experience of “fear” but not “anxiety.” Over the years, it has become increasingly clear that tone-shock pairing in rodents is insufficient to understand the complete phenomenology of fear (LeDoux, 2014). However, as we will discuss below, the neural circuits involved in tone-shock conditioning are implicated in anxiety disorders by other assays of fear- and anxiety-relevant behaviors.

We use the undifferentiated term “fear and anxiety” to refer to the collective set of affective states associated with distress in anxiety disorders, which often are experienced in combination with persistent worry, physiological changes, and avoidance behavior. The intentional grouping of these terms can lead to clearer insights into the heterogeneity of anxiety disorders by avoiding a false dichotomy and incorporating relevant information from a variety of sources. Ultimately, this approach promises to allow scientists to “carve nature at its joints” and better parcellate the heterogeneity within the experience of fear and anxiety.

## Heterogenous presentation of anxiety disorders

The current categorical approach to defining anxiety disorders raises challenges to advancing our understanding of the disorder. The DSM-5 currently distinguishes 12 different anxiety-related disorders. Each disorder is characterized by excessive and persistent worry but differentiated by specific diagnostic criteria and a diverse range of triggering stimuli. Ultimately, categorical diagnoses rely on self-reported symptom profiles that are expressed across a variety of contexts that are not explicitly linked to biology or treatment efficacy. Structured clinical assessments (First & Caban, 2010) and efforts to develop empirical taxonomies (Kotov et al., 2022) can help to address these issues, but they have yet to be adopted into standard clinical settings. Thus, patients with different disorders can respond to the same treatment (“one-to-many”), whereas patients with the same disorder may not (“many-to-one”).

**Fig. 1:**
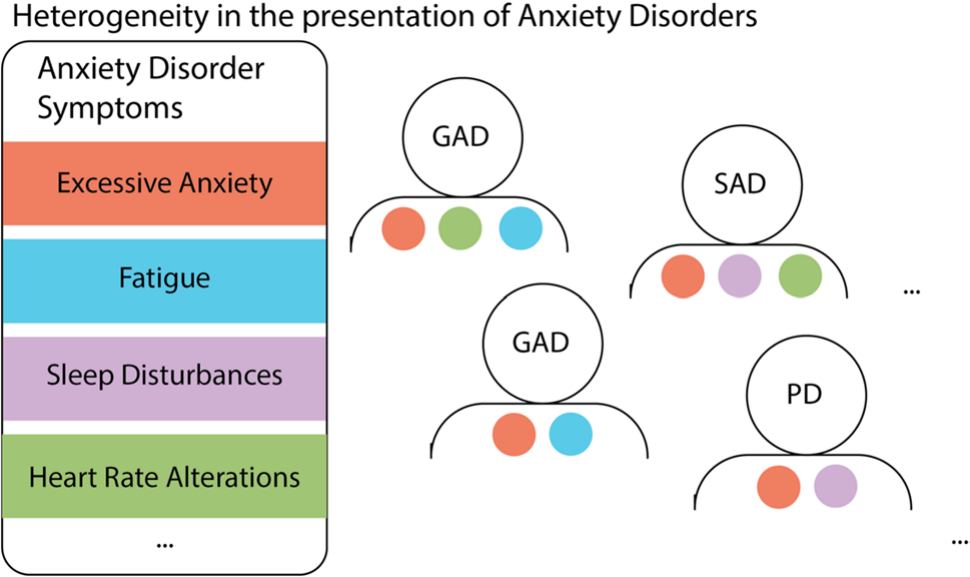
Heterogeneity in the presentation of anxiety disorders. A schematic depicting a subset of anxiety disorder symptoms (left) and how a subset of patients can present with some but not all symptoms. Each patient can have a different symptom profile that can be shared with patients with distinct diagnoses. GAD = generalized anxiety disorder; SAD = social anxiety disorder; PD = panic disorder

Importantly, different anxiety diagnoses can share symptom profiles. For example, patients with social anxiety and patients with agoraphobia, two distinct anxiety disorders as defined by the DSM-5, may show the same signs of distress (e.g., racing heart, sweating, nausea, shortness of breath) (Fig. 1) in response to different stimuli. As such, it is difficult to distinguish patients based on their overt expression of anxiety. This could present a problem in selecting optimal treatments. If a treatment is targeted at brain systems required for the symptom, and not specifically linked to the source of the anxiety itself, the same treatment could be equally effective in two individuals with different disorders.

In contrast, anxiety disorders can be highly heterogeneous in their presentation within a diagnostic category. There is substantial variability in clinical presentation across individuals (Altemus et al., 2014; Galatzer-Levy & Bryant, 2013; Lenze & Wetherell, 2011) (Fig. 1), and when using the current DSM-5 guidelines, there are nearly endless combinations of symptom profiles that may qualify for the same anxiety disorder diagnosis. This symptomatic heterogeneity suggests a corresponding heterogeneity in the underlying neural circuitry of these symptoms, which has likely contributed to the ineffectiveness of one-size-fits-all treatment approaches. Thus, patients who present with the same disorder may not respond to the same treatment.

In summary, anxiety disorders are clearly heterogeneous and multifaceted. As such, our approach to understanding, diagnosing, and ultimately treating anxiety disorders must be as well (Akil et al., 2010). The development of new treatments will require acknowledging that relationships between biology and anxiety disorder symptoms can be both “one-to-many” and “many-to-one.”

## Identifying the brain regions involved in anxious temperament and anxiety disorders

Perhaps unsurprising given the heterogeneity of anxiety disorders, human neuroimaging research has not consistently identified a single region as the sole contributor to feelings of fear and anxiety. Instead, studies have identified a distributed neural circuit that is associated with many aspects of anxiety disorders. This distributed fear and anxiety circuit includes a broad array of subcortical and cortical structures, including the amygdala, bed nucleus of the stria terminalis (BST), hypothalamus, hippocampus, anterior cingulate cortex, insula, and the medial prefrontal cortex (Chavanne & Robinson, 2021; Etkin & Wager, 2007; Shin & Liberzon, 2010). Although a full review of the specific contribution of each of these regions is outside of the scope of this review, it is important to note that the brain is not equipotent, and each of these regions perform distinct computations in concert to give rise to the holistic experience of fear and anxiety.

Importantly, these studies and others emphasize a relationship between acute anxiety, dispositional (or trait) anxiety, and anxiety disorders. For example, many of the same brain regions that have been implicated in pathological anxiety also are activated during paradigms designed to elicit anxiety in control subjects (Chavanne & Robinson, 2021), suggesting that anxiety disorders may arise from maladaptive application of these systems to daily life. In support of this, individuals with higher levels of dispositional anxiety show heightened response to stressors and are more likely to develop anxiety disorders (Clauss & Blackford, 2012; Hengartner et al., 2016; Shackman et al., 2016). These findings suggest that similar neural processes contribute to both pathological and nonpathological anxiety. Furthermore, they highlight that studying pathology is not essential to gain insights into the function of these regions in anxiety disorders.

**Fig. 2:**
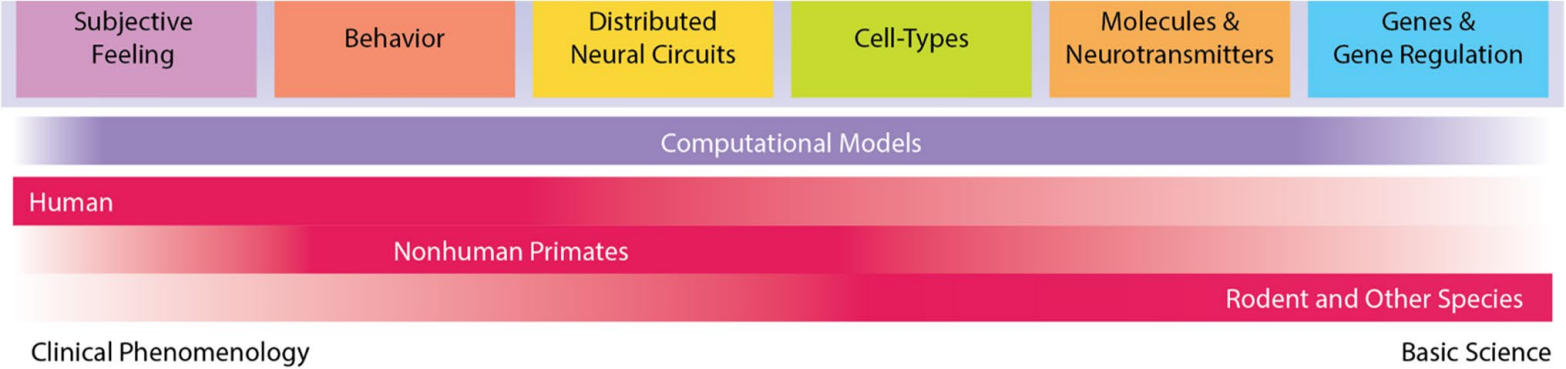
Anxiety research spans many disciplines. Animal models provide a framework for examining the neurobiology that gives rise to anxiety and fear, and unique animal models are better suited to answer specific questions at different levels of analysis. Computational models offer an opportunity to bridge the gap between different models and levels of analysis

Research designed to understand the specific contributions of each brain region implicated in fear and anxiety will require animal models (Bale et al., 2019). Animals cannot reliably report their subjective experience, ultimately necessitating additional measures and the study of nonpathological anxiety. In humans, subjective feelings of fear and anxiety emerge from or are related to behaviors, distributed neural circuits, specific cell types, molecules and neurotransmitters, and genes and gene regulation (Grogans et al., 2023) (Fig. 2). Disruptions in any of these systems could lead to extreme feelings of fear and anxiety, and as such, optimal treatments will require comprehensive approaches that target each of these levels of analysis.

Next, we highlight the multidisciplinary animal modeling approaches that have contributed to our understanding of the neurobiology of anxiety disorders and argue that the next generation of translational anxiety research must work across species, levels of analysis, and disciplines to work toward more effective treatments.

## Decomposing the distributed neural circuits that underlie threat responding

Animal models are uniquely well-suited to investigate causality and aid in our understanding of the threat-relevant computations within specific brain regions. The ability to detect and respond to threats is largely conserved across species, and the ubiquity of flight and freezing behaviors in threatening contexts highlights their adaptive success in threat-responding (Roelofs, 2017). Cross-species similarities in threat detection and responding form the basis of translational efforts to understand the neurobiology of fear and anxiety in animal models. Researchers use a wide variety of ethologically relevant paradigms to assess threat-responding in rats and mice—the most commonly used animals in neuroscientific research (Calhoon & Tye, 2015; Haller et al., 2013; Hickman et al., 2017). Although animals cannot report their experience, focusing on ethologically-relevant threat responding is supported by pharmacological studies that show responsiveness to drugs that decrease anxiety in humans (Borsini et al., 2002) (although see Box 1). Together, this body of work supports the notion that an understanding of the neurobiology of threat perception and responding in animals can guide our understanding of the mechanisms that give rise to extreme and chronic anxiety in humans.

Rodent research confirms observations in humans that threat-responding is instantiated across multiple threat-relevant brain regions that act in concert or competition with each other to initiate adaptive defensive behaviors. Similar to humans, this network of threat-responsive regions includes the amygdala, BST, hypothalamus, hippocampus, prefrontal cortex (PFC), and periaqueductal gray (PAG), among other regions (Adhikari, 2014).

Rodent models can extend human research to identify the precise neurobiological mechanisms that underlie specific threat-responses in certain contexts. For example, in studies of tone-shock conditioning in rodents, the amygdala initiates freezing behavior in response to a conditioned tone stimulus. Learning is thought to occur in amygdala neurons across the basolateral nucleus of the amygdala (BLA) and the central nucleus of the amygdala, lateral part (CeL). These regions induce freezing via projections to the central nucleus of the amygdala, medial part (CeM). The CeM, in turn, inhibits local interneurons in the ventrolateral PAG (vlPAG), which through feed-forward inhibition results in excitation of neurons in the medulla that initiate freezing through spinal cord and forelimb muscles (Tovote et al., 2016). This represents decades of research, designed to understand a specific circuit that initiates fear- and anxiety-related freezing in a specific learned context, and highlights multiple places where insults and vulnerabilities could lead to increased risk for anxiety disorders.

**Fig. 3:**
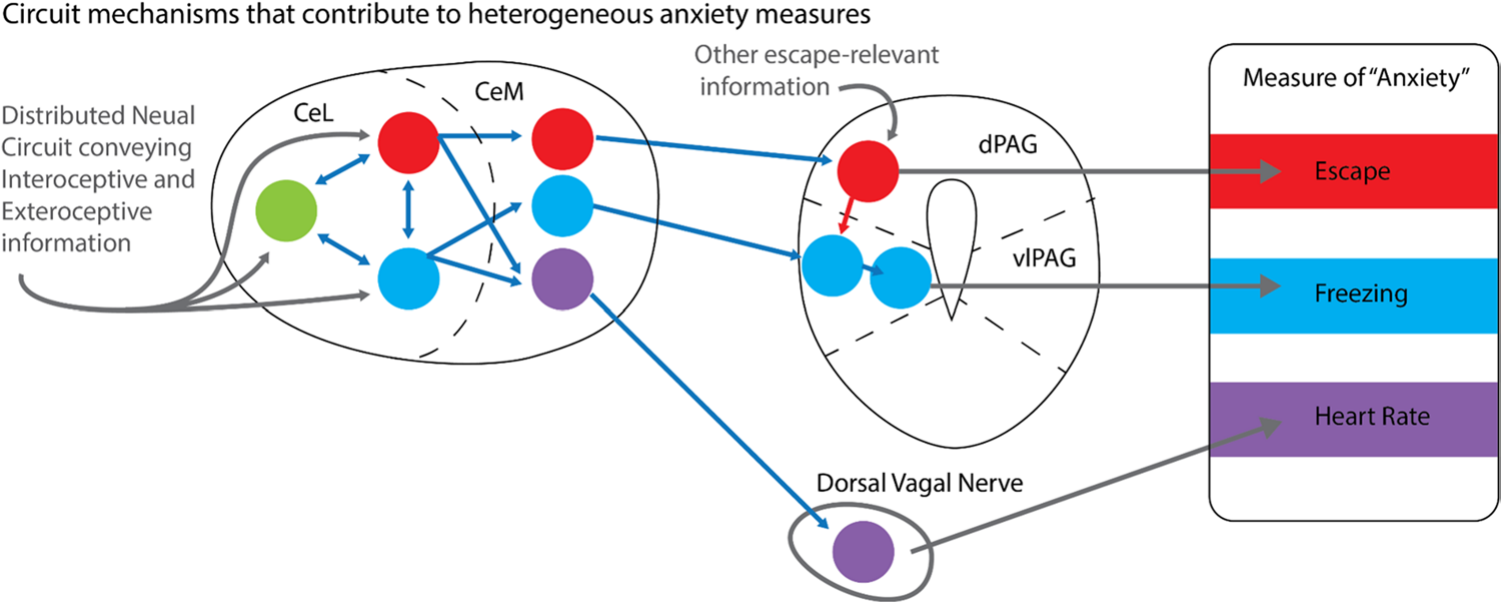
Diagram of circuit mechanisms that can contribute to the heterogeneity of fear and anxiety measures. Mutually inhibitory networks in CeL can trigger distinct populations of CeM output neurons that project to PAG and other downstream regions (such as the dorsal vagal nerve) to initiate the varied responses that are used in animal studies of fear and anxiety. CeL = central amygdala, lateral; CeM = central amygdala, medial; dPAG = dorsal periaqueductal gray; vlPAG = ventrolateral periaqueductal gray

Importantly, animal models have shown that not all threats are processed the same way in the brain. The neural responses induced by threat are highly unique to the stimulus (Sanford et al., 2017), and the same cells or brain regions can be implicated in the execution of a variety of behaviors (Deng et al., 2016). For example, while the CeM to PAG projections are critical for freezing during tone-shock conditioning (Tovote et al., 2016), distinct projections from the medial superior colliculus to PAG initiate escape from a looming shadow (Evans et al., 2018). Specifically, dorsal PAG neurons can be activated by mSC projections, which excite the vlPAG interneurons to inhibit freezing and facilitate escape behaviors (Tovote et al., 2016). These data highlight partially overlapping neural circuits in both freezing and escape behaviors (Fig. 3) and highlight the fact that not all behaviors can be implemented at the same time—an animal cannot escape while freezing.

Thus, competition within and across fear- and anxiety-related brain circuits is critical for selecting the appropriate emotional response (Holley & Fox, 2022). This competition is not unique to the PAG. For example, distinct sets of mutually inhibitory cells in the CeL compete to determine the appropriate response (Fadok et al., 2017; Isosaka et al., 2015). Specifically, stimulation of somatostatin (SST) and corticotropin releasing hormone (CRH) positive cells can initiate freezing and escape, respectively. Because these cells are mutually inhibitory, this provides a potential mechanism for competition between competing responses. The microcircuitry of the CeA allows for this region to induce multiple, distinct, survival-relevant behaviors (Holley & Fox, 2022; Moscarello & Penzo, 2022).

Together, these data reveal how different types of cells collaborate and compete to initiate threat-responding across multiple contexts. This level of understanding is only possible with the help of animal models, which have illuminated a complex network of threat-responsive and heterogenous brain regions and suggest many insights into potential points of intervention to treat anxiety disorders. For example, disruption at various cell types or multiple nodes within the circuit discussed above could lead to altered freezing behaviors, underscoring the limitations of a “one-size-fits-all” treatment for anxiety disorders.

These data highlight two major challenges for translational research: 1) translating findings from rodents to humans, and 2) expanding and identifying the appropriate animal models that are mostly likely to be relevant to understanding the heterogeneity of fear and anxiety in humans.

## Translating neuroscience findings from animals to humans

Animal models can be used to develop testable hypotheses about the mechanisms of fear and anxiety in humans. Ultimately, because we cannot say with certainty how an animal is feeling, work in humans must validate the role for specific microcircuits in the subjective experience of anxiety (LeDoux & Brown, 2017; LeDoux & Pine, 2016). For example, whether the same cells activated during freezing in a tone-shock paradigm are the same cells that contribute to subjective feelings of anxiety in humans is uncertain. More generally, the causal contribution of a specific cell type during a particular assay in a rodent does not imply that human fear and anxiety relies on this same circuit. To this end, experiments that: 1) leverage defensive paradigms adapted from animal research, and 2) build on the known mechanisms of anxiety-related behavior in rodents will be critical for developing targeted interventions for anxiety-related psychopathology.

Research in this area is ongoing and has begun to demonstrate the correspondence between humans and nonhuman animals. For example, theories of context-dependent defensive behavior in rodents have been instrumental in motivating human work focused on the threat-imminence continuum, which when used in animals elicits a diversity of threat-responsive behaviors and neural activation patterns based on the proximity of the threat (Blanchard et al., 2011; Fanselow, 1994). In a virtual avoidance paradigm, participants avoid a virtual predator that can chase, capture, and cause pain in the form of shocks (Mobbs et al., 2007). Mirroring animal findings (Evans et al., 2018; Kim et al., 2018), the patterns of brain activity in humans engaged in this task fluctuate with the proximity and likelihood of the threat. Specifically, more frontal regions are engaged during the first encounter with the virtual predator, when the threat is more distal. There is a shift toward increased activity in the midbrain PAG when threat is near and the subject engages in avoidance strategies (Mobbs et al., 2007). This paper represents a thoughtful extension of rodent work into human fear and anxiety and demonstrates the utility of a videogame-like assay for translating animal assays to humans.

Because the cells involved in fear and anxiety are distributed across the brain, methods that predict symptoms solely on a single brain region will not likely yield clinically relevant findings. However, researchers can test hypothesized functional relationships between regions by examining the patterns of BOLD activation across multiple brain regions and stimuli that elicit different adaptive behavioral responses (i.e., using fMRI measures of functional connectivity). For example, animal studies demonstrate strong reciprocal projections between the Ce and BST (Oler et al., 2017) that are involved in sustained anxiety (Asok et al., 2018). Functional connectivity in rhesus monkeys shows that individual differences in a stable and heritable anxiety phenotype were associated with rsfMRI measures of Ce-BST functional connectivity (Fox et al., 2018), suggesting that these projections are relevant to human fear and anxiety***.*** This hypothesis, and other similar hypotheses that implicate projections from one region to another, can be tested in humans by using rsfMRI, but has been hindered by the fact that these regions are small, and many scanners lack the temporal and spatial resolution needed to parse these microcircuits. Ongoing work using high-field fMRI has begun to better assess the connectivity between these regions with increased anatomical precision using high-resolution imaging at 7 T (Hofmann & Straube, 2021; Torrisi et al., 2019; Weis et al., 2019). Additional methods using lower resolution imaging have been used to parcellate these small subregions, including differentiating the amygdala based on its connectivity patterns with other regions and hand-drawing ROIs (Pedersen et al., 2020; Sylvester et al., 2020; Tillman et al., 2018). Thus, high resolution may not be required to test projection-specific hypotheses about limbic microcircuits derived from animal models. This avenue of research promises to identify projection-specific contributions to fear and anxiety and is ripe for additional research.

**Fig. 4:**
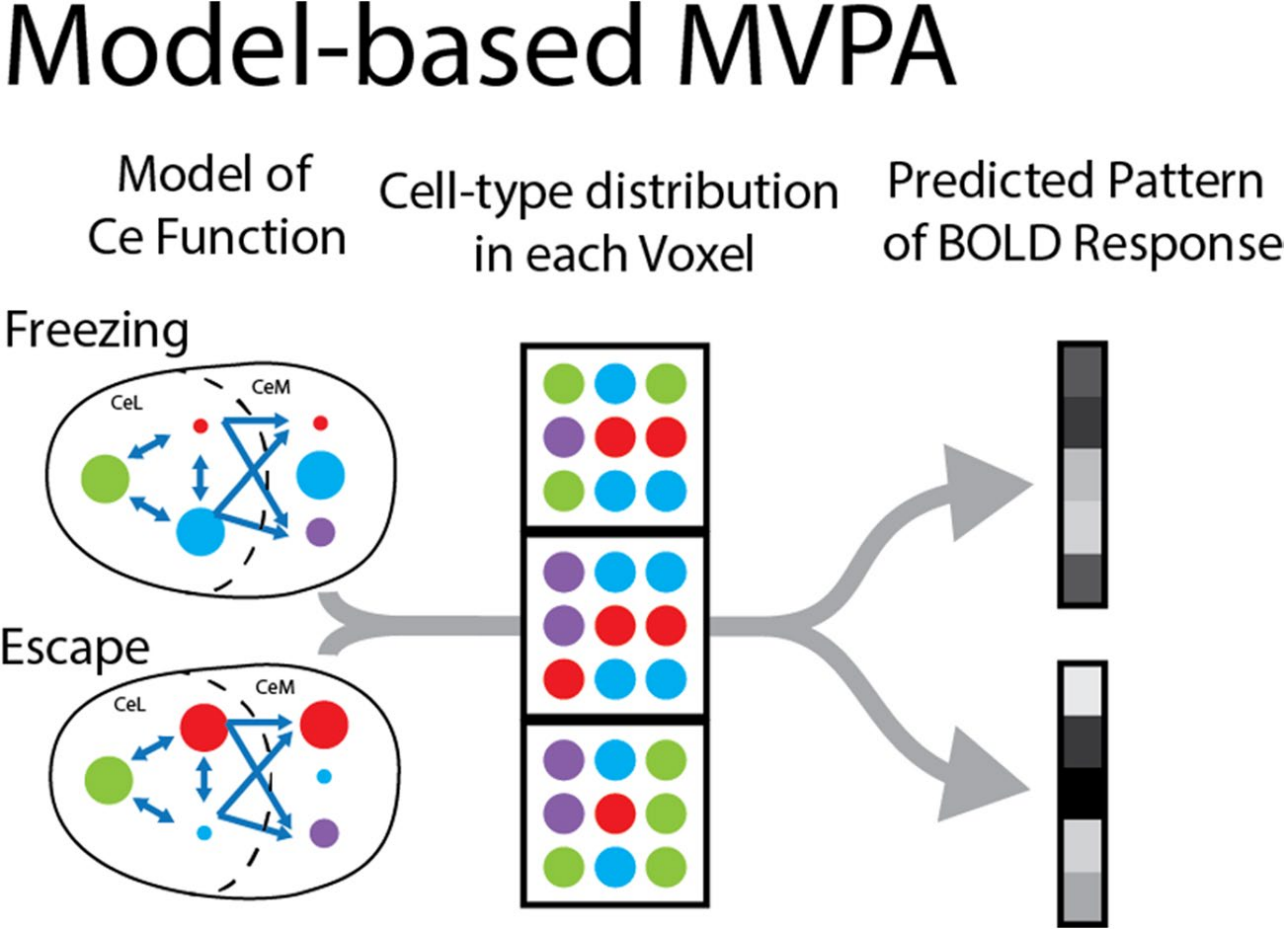
Model-based MVPA. A schematic of how different models of Ce function based on experiments in rodents can make predictions about the pattern of activation in human fMRI studies. Because the distribution of cell types contributing to different behaviors are not uniformly distributed across voxels, different behaviors are hypothesized to be associated with differences in the pattern of BOLD response across Ce voxels. Ce = central amygdala

In addition to projection-specific hypotheses, the within-region heterogeneity in specific brain regions poses a challenge for conventional neuroimaging methods used in human subjects. Neuroimaging voxels reflect a diverse population of hundreds of thousands of neurons (Logothetis, 2008) and are not precise enough to dissect the specific contributions of distinct neural cell types. Yet, as outlined above, within the amygdala there are multiple populations of mutually inhibitory cell-types. Consequently, the measured BOLD signal constitutes the activity of competing microcircuits. Critically, different cell types are not uniformly distributed within regions (Beyeler et al., 2018; McCullough et al., 2018), and different voxels likely reflect distinct compositions of Ce cell types. Because of this within-voxel heterogeneity, multivoxel pattern analysis (MVPA) can provide an approach for testing hypotheses relating to distinct functional patterns associated with different cell-types being differentially involved in specific processes in humans (Norman et al., 2006). More specifically, researchers translating findings from rodent models can leverage this across-voxel heterogeneity to design experiments that might reveal distinct processes within a region. For example, if each CeL voxel contains a distinct mixture of SST and CRH neurons, the pattern of activity should reflect these mutually inhibitory local circuits. Based on the mutually inhibitory SST and CRH neurons in Ce reviewed, we hypothesize that Ce patterns would predict the use of different defensive strategies (i.e., freezing vs. escape) (Fig. 4). As discussed below, this work can form the foundation for neurobiologically derived computational models.

More generally, MVPA can detect patterns of activity across multiple voxels, which occur independently of the expression of measurable behaviors (Polyn et al., 2005). This technique therefore can be used to infer cognitive states of the subject and could be used to distinguish brain states that contribute to the subjective feeling of fear and anxiety from those that represent other aspects of the task (e.g., physiological responses)—a crucial step in translational work. Using this approach, researchers have identified underlying neural dissociations between the subjective feeling of fear and its physiological correlates (i.e., skin conductance), emphasizing that the experience of fear is not solely the result of fear-related physiological activity (Taschereau-Dumouchel et al., 2020). We suggest that MVPA can be used to identify patterns of brain activation associated with the subjective experience of fear and anxiety, which provides a path to understand the relationship between defensive circuits identified in animal models and the experience of fear and anxiety in human populations.

## Capturing fear and anxiety across species: The value of nontraditional animal models

Not all aspects of anxiety disorders are equivalently modeled in all nonhuman animals, and not all anxiety-relevant brain regions are equivalently conserved across species. It remains possible that even a complete understanding of the brain of a standard laboratory mouse will be insufficient to recapitulate similar circuits in humans. Thus, it is critical that we consider additional species that are better suited to understand the variety of biological processes that give rise to specific symptoms implicated in the heterogeneity of human anxiety. Different animal species possess their own unique characteristics that make them appropriate to model unique aspects of fear and anxiety.

Other rodent species may be better suited to studying specific aspects of fear and anxiety in nonhuman animal models beyond the most commonly used laboratory mice and rats (*Mus musculus* and *Rattus norvegicus*, respectively) (Hickman et al., 2017). For example, in humans there are well-defined relationships between the menstrual cycle and anxiety (Kuehner & Nayman, 2021; Yen et al., 2020). Standard laboratory rodents have an estrous cycle (Kundakovic & Rocks, 2022), which has been shown to influence fear learning and extinction (Milad et al., 2009; Zeidan et al., 2011). While the rodent estrous cycle is similar to the human menstrual cycle, there are several key differences. Primarily, estrous cycles do not include the cyclical shedding of the uterine lining in the absence of pregnancy (menses), which is under the control of the hypothalamic pituitary gonadal (HPG) axis (Hall, 2019). Menstrual irregularities, which are mediated by the HPG axis, are associated with increased depression symptoms (Bisaga et al., 2002; Toffol et al., 2014), necessitating a different model to better understand the relationship between the menstrual cycle and anxiety in humans. Additionally, estrous and menstrual cycles differ in their lengths (4–5 days in rodents vs. ~28 days in humans). Animal models that more accurately reproduce the timing of the menstrual cycle are important, because the effects of gonadal hormones are often temporally dependent and can be long-lasting (Galea et al., 2017). Recent work has demonstrated that the spiny mouse (*Acomys cahirinus*) has a menstrual cycle (Bellofiore et al., 2017), providing a unique animal model in which to understand hormone-related psychopathological symptoms in the laboratory (Bellofiore et al., 2019).

Another limitation of standard laboratory rodents is the condensed developmental timeline. In humans, there are childhood risk-factors for the development of anxiety disorders (Cabral & Patel, 2020), as well as a period of increased incidence during adolescence (Beesdo et al., 2007). Laboratory mice are considered adults by the age of postnatal Day 60, with a brief adolescent period lasting approximately 3 weeks (Drzewiecki & Juraska, 2020). Therefore, experimental setups that require repeated exposure or training cannot be performed in standard laboratory rodents. Again, other rodent models may be better suited to understanding these aspects of fear and anxiety. For example, the California mouse (*Peromyscus californicus*) has an extended adolescent period and reaches adulthood at postnatal Day 90, making this animal well-suited to understanding the changes that increase adolescent-onset anxiety (Wright et al., 2023).

Studying the longer-term changes that occur throughout childhood that put an individual at risk for anxiety disorders will require animal models that have a protracted developmental timeline. To this end, studying nonhuman primates, such as rhesus macaques (*Macaca mulatta*), can be particularly useful. Researchers have established similarities between early-life anxious and inhibited temperament across humans and rhesus macaques (Fox & Kalin, 2014; Kenwood & Kalin, 2021), which allow for the study of the neurobiology that gives rise to the emergence of anxiety disorders in humans. This work has revealed similarities between humans and rhesus macaques in the distributed brain network associated with individual differences in temperament (Fox & Shackman, 2019; Oler et al., 2010), identification of brain regions that likely mediate the inherited aspects of temperament (Fox, Oler, Shackman et al., 2015a; Fox et al., 2018), and initial suggestions about the molecular (Fox et al., 2019; Kalin et al., 2016; Kenwood, Souaiaia et al., 2023b; Kovner et al., 2020) and genetic (Fox et al., 2021) mechanisms that underlie the early-life risk to develop anxiety disorders. This work has been instrumental to drawing attention to the central extended amygdala, encompassing the Ce and the BST in anxiety and anxiety disorders (Fox et al., 2018; Fox, Oler, Tromp, et al., 2015b; Fox & Shackman, 2019).

Finally, when considering cross-species studies, researchers must consider how the biological processes underlying threat-processing have been shaped by species-specific evolutionary pressures over millions of years. The brain circuits that underlie fear and anxiety in humans have continued to evolve and have been incorporated into a highly integrated network of brain regions. This is evidenced by increasingly specialized sensory cortices (Kaas, 2008), an expanded prefrontal cortex (Donahue et al., 2018; Smaers et al., 2017), as well as alterations in the organization and cellular composition of individual brain regions (Chin et al., 2023; Gibbs et al., 2007; Krienen et al., 2020; Schmitz et al., 2022). These evolutionary changes likely create unique aspects in cognitive processing that may influence the experience of fear and anxiety (Pine et al., 2021). As such, it is critical that we do not take brain-behavior homology for granted.

Nonhuman primates (NHPs) diverged from humans much more recently than rodents (Nei et al., 2001; Stewart & Disotell, 1998), thus providing an important translational model to verify and extend brain-behavior homology as it relates to fear and anxiety (Campos et al., 2023; Fox & Shackman, 2019; Roberts & Clarke, 2019). Humans are most closely evolutionarily related to other simian species, including Old World Monkeys and New World Monkeys, which diverged ~30 and ~50 million years ago, respectively (Stevens et al., 2013; Yang et al., 2021a). Macaques (Old World) and marmosets (New World) are the two most commonly used NHP species in anxiety research (Ausderau et al., 2023); each have a distinct utility for studying aspects of anxiety and fear based on their ethology and ecological niche (Capitanio, 2017; Capitanio & Emborg, 2008; Gunnar et al., 2015; Kalin & Shelton, 2003; Machado & Bachevalier, 2003; Miller et al., 2016; Mitchell & Leopold, 2015; Okano, 2021; Saito, 2015). NHPs have been leveraged to confirm the evolutionary conservation of brain-behavior relationships for many threat-relevant regions (Agustín-Pavón et al., 2012; Fox, Oler, Shackman et al., 2015a; Izquierdo et al., 2005; Kalin et al., 2004, 2007; Kenwood & Kalin, 2021; Machado & Bachevalier, 2008; Prather et al., 2001; Shiba et al., 2015). They also enable investigation of the role that these regions play in complex socioemotional behaviors across the lifespan, including adolescent development, life-long plasticity, and social behaviors (Alisch et al., 2014; Bliss-Moreau et al., 2017; Jacobs et al., 2023; Kovacs-Balint et al., 2023; Wellman et al., 2016).

Perhaps most obviously, NHPs are uniquely well-suited to model cognitively-elaborated aspects of threat processing associated with the evolutionary expansion of the prefrontal cortex (García-Cabezas et al., 2019; Öngür & Price, 2000; Roberts & Clarke, 2019). In addition to being implicated in threat processing (Kenwood et al., 2022), the PFC plays an important role in attention, cognition, and decision-making (Miller et al., 2002). The primate PFC is a functionally heterogeneous region, which is largely, but not fully, homologous across humans, rhesus, and marmosets (Amiez et al., 2023; García-Cabezas et al., 2019; Kenwood et al., 2022; Preuss & Wise, 2022; Tian et al., 2022). Studies of threat-relevant behaviors have implicated NHP PFC subregions, including the orbitofrontal cortex (OFC), dorsolateral PFC (dlPFC), and medial PFC (mPFC) (Birn et al., 2014; Fox, Oler, Shackman et al., 2015a; Roberts & Clarke, 2019). Importantly, these PFC regions do not have clear homologies within the rodent PFC (Laubach et al., 2018), and even when they do, they often have different patterns of connectivity (Amaral & Price, 1984; Ghashghaei & Barbas, 2002; Price, 2003). As such, the contributions of frontal regions to fear and anxiety cannot be readily modeled in rodents, and understanding the heterogeneity of anxiety disorders in humans will likely require NHP models.

NHPs have been used to uncover the role of specific frontal regions in threat processing, highlighting a heterogenous role for OFC subregions in various aspects of threat-responding and threat-related decision making. In macaques, lesioning the entire OFC decreases freezing in potentially threatening contexts (Fox et al., 2010; Izquierdo & Murray, 2004, 2005; Kalin et al., 2007; Machado & Bachevalier, 2008). These effects are thought to be mediated by connections with subcortical regions, with lesions of the OFC or fibers through the OFC leading to corresponding decreases in freezing and BST metabolism (Fox et al., 2010; Kenwood, Oler et al., 2023a). However, in both marmosets and macaques, inactivation or excitotoxic lesions of specific OFC subregions can have the seemingly opposite effect (Agustín-Pavón et al., 2012; Clarke et al., 2015; Pujara et al., 2019; Rudebeck et al., 2013). For example, in an approach-avoidance conflict task, pharmacological inactivation of area 11 of the OFC disrupts punishment-associated memories. Marmosets with area 11 inactivation increased their avoidance of punishment-related stimuli, even in the absence of explicit punishment, suggesting an increased level of anxiety (Clarke et al., 2015). These findings reinforce the heterogeneity of behaviors that are termed “anxiety” and converge with other research showing a more general role for specific OFC regions in different aspects of value-learning and stimulus-relationship outcomes (Wallis, 2012). Together this points to the need for computational models to link distinct tasks across species. Although there is much work to be done, these studies demonstrate the value of NHPs for studying specific aspects of threat-processing because of their recent evolutionary divergence and cortical similarity to humans.

These are but a few examples of how different species can have distinctive characteristics based upon their unique behaviors, reproductive physiology, evolutionary history, social/family structures, etc. All of these differences can make an animal suitable or unsuitable for investigating particular aspects of fear and anxiety. In short, no one species is ideal, and translational neuroscience is best suited by drawing on the wide variety of species in the animal kingdom (Kenkel et al., 2021; Lima & Dill, 1990; Maximino et al., 2015; Preuss, 2019; Shannonhouse et al., 2014). To this end, selecting the most appropriate animal models for gaining insight into select aspects of human anxiety is critical. To prioritize relevant animal studies and species selection, researchers must thoughtfully engage in reverse translation (i.e., use evidence from studies of human populations to guide animal research). Because of the heterogeneous presentation of anxiety in humans, researchers will need to identify specific aspects of the disorder that are best modeled in different species. Basic neuroscience approaches should be combined with ethologically relevant assays to identify the biological mechanisms that underlie that aspect of fear and anxiety. Finally, results from these studies will inform translational research by guiding the development of computational models and developing hypotheses based on animal work that can be tested in humans.

## Developing new approaches to understanding fear and anxiety: A role for computational models

Developing additional, ecologically relevant assays and incorporating additional species presents a new set of challenges for interpreting findings in relation to human fear and anxiety. To overcome these challenges, we propose that researchers use computational models in combination with targeted empirical studies. Although underutilized in studies of fear and anxiety, theory-driven computational modeling can guide the development and interpretation of new paradigms, and enable cross-species integration (Huys et al., 2021). Computational models designed to model underlying processes that mediate threat perception and the underlying mechanisms that lead to different feelings, behaviors, and symptoms. These models can be used to develop new hypotheses about the precise role that specific brain cells are playing in anxiety and begin to shed light on the distributed neural circuit associated with fear and anxiety. This approach has been extremely successful in uncovering computations associated with reward learning in coordinated cross-species efforts (e.g., prediction errors coding in the VTA; Dabney et al., 2020; D’Ardenne et al., 2008; Jeong et al., 2022; Schultz et al., 1997) and could be applied to fear and anxiety. Recent efforts have begun to develop novel computational models derived from 1) ethology (Mobbs et al., 2021), 2) the statistics of the environment (Pulcu & Browning, 2019), and 3) the underlying functional neurobiology of brain regions (O’Reilly et al., 2019).

Ethologically derived computational models are designed to develop hypotheses about the underlying computations that drive behavior across varying contexts. These models are built upon studies of animal behavior, which have identified parameters of the environment that signal the nature of a potential threat to guide adaptive responses. Computational models extend these findings by proposing dynamic processes that can explain behavior based on varied sources of information (e.g., distance, probability, type of threat, etc.). For example, Mobbs and colleagues have proposed model-based policies that are derived from distributed brain activation to guide behavioral selection across the threat imminence gradient (Mobbs et al., 2020). The neural computations that occur in response to threat will vary across spatial and temporal parameters. For instance, an animals’ decision to freeze or escape is based on spatial and temporal aspects of the threat, as well as internal underlying states of the animal and the environment (Holley & Fox, 2022). Importantly, these underlying states cannot be inferred from behavior alone (Box 1). Ethologically based modeling approaches allow us to begin to disentangle the underlying computations that contribute to the execution of adaptive survival behaviors (Mobbs et al., 2020).

Computational models derived from the statistics of the environment provide a complementary approach to understanding fear and anxiety. Like ethologically driven models, these models are designed to be more precise about the nature of the threat, moving beyond imprecise language and providing explanations that extend beyond a particular context. For example, although the term “uncertainty” has been compellingly associated with the experience of anxiety (Grupe & Nitschke, 2013), there is a lack of consistency with how uncertainty has been defined. Computational models have targeted specific environmental parameters that fall under the umbrella of “uncertainty.” Uncertainty-related parameters include: the unknowable probability of an event (Lawrance et al., 2022), variance in the outcome of an action (Browning et al., 2015), and the evolving probability that an event will occur given that it has not already happened (Holley, personal observation, 2023). Each of these factors could be termed “uncertainty,” but in computational terms, each is an independent and dissociable factor that can be independently manipulated to increase fear and anxiety. For example, during an aversive learning task, Browning et al. demonstrated that patients with anxiety failed to adapt to a changing environment when action-outcome relationships become increasingly variable. Disambiguating different statistical features that contribute to uncertainty provides an avenue to understand how the brain encodes these distinct aspects of “uncertainty” and how these computations contribute to the subjective experience of fear and anxiety.

Finally, computational models derived from the underlying neurobiology provide a “bottom-up” approach to understanding fear and anxiety. These models are built on our current understanding of the brain and uses observations of neuronal firing patterns in rodents to make predictions about how these neurons contribute to complex processing in humans. This approach has been successful in identifying a grid-like code, based on research in mice, for abstract concepts in humans. Building on the discovery of grid cells in mice (Fyhn et al., 2008; Rowland et al., 2016), researchers developed computational models of how grid-cell firing would manifest in fMRI data during virtual egocentric exploration in humans (Doeller et al., 2010). Critically, this computational model was applied to demonstrate grid-like coding of complex conceptual information as humans performed complex tasks (Constantinescu et al., 2016; Park et al., 2021). We encourage researchers to reflect on these published works, because they provide an excellent example of how neurobiologically derived computational models can be used to shed light on human-specific experiences, as will be required to understand fear and anxiety.

A neurobiologically inspired computational approach could be extended to population coding in the Ce. In the Ce, researchers have demonstrated: 1) Ce metabolism that is not specific to a particular threat-response (Shackman et al., 2013); 2) distinct neurons in CeM that are sufficient to induce different aspects of a threat response (Viviani et al., 2011); 3) neurons in CeL that project to CeM to induce threat responses (Haubensak et al., 2010); and 4) mutually inhibitory populations of CeL neurons that can elicit different threat responses when stimulated (i.e., freezing and escape) (Fadok et al., 2017). Although no studies have specifically investigated mutually inhibitory networks in Ce using fMRI, this area is ripe for study and could begin using the MVPA framework outlined above. Researchers have begun to propose computational models in which the CeL is integrating across different threat-relevant features and performing computations to select the response that is expected to be most adaptive (Holley & Fox, 2022; Moscarello & Penzo, 2022).

Together, computational models will be critical for advancing our understanding of fear and anxiety by making predictions that are not specifically related to the threat assay used or behaviors measured. Moreover, computational models can be leveraged to understand the many presentations of anxiety disorders. These models provide a framework for understanding how the same neurobiological mechanism can result in heterogeneous presentations and/or how the same presentation of anxiety can result from multiple underlying mechanisms (e.g., via different model-based policies or CeL computations). This enables researchers to correlate and manipulate specific parameters to identify the neural systems that underlie threat-relevant computations across varied behaviors and anxiety assays (which can be less specific, see Box 1). Although computational models have been underutilized in the context of fear and anxiety, ethologically, statistically, and neurobiologically derived computational approaches promise to identify specific computations instantiated in the varied cells and circuits within the distributed anxiety network that can guide the development of new treatment strategies.

## Conclusions

There is substantial heterogeneity in the presentation of anxiety disorders. This heterogeneity is reflected in the distributed neural mechanisms that can contribute to fear- and anxiety-related behavior and the lack of one-size-fits all treatments. Overcoming this complexity and take the first steps toward developing more effective treatments, will require 1) translation of preclinical basic neuroscience research in rodents to test predictions about human anxiety, 2) reverse translation of clinical observations in humans using multiple animal models, including NHPs and nontraditional rodent species; 3) the development of computational models that can guide theory construction. A refined understanding of the brain circuits that give rise to anxiety and fear is a critical next step and is a prerequisite for identifying specific behavioral or pharmacological treatments that optimally treat anxiety disorders. Although daunting, we have demonstrated that this work is possible, highlighted ongoing efforts that have been successful, and suggested specific experiments that would begin to address these challenges. To this end, we strongly encourage dialogue and collaboration between basic neuroscientists and clinicians to facilitate translation and reverse translation designed to maximize the impact of future studies to understand the biological bases of heterogeneity in anxiety disorders.

## BOX 1: Considerations for interpreting measures of fear and anxiety

The translational study of fear and anxiety requires the study of individual humans and animals through the lens of a few measures in a limited set of contexts. Anxiety assays by necessity measure acute and context-specific behaviors. This stands in contrast to the experience of fear and anxiety in individual suffering from anxiety disorders, which arise from heterogeneous contexts and produce heterogeneous responses. We highlight how this applies to animal models of fear and anxiety to exemplify the complexity of this problem and why translational work must incorporate multiple measures.

### Behaviors do not exclusively represent a single, affective state

Animal models infer anxiety by examining observable behaviors, such as locomotion or freezing. Animal models are critical for demonstrating causality in neuroscience (Bale et al., 2019). However, many have questioned the validity of commonly used animal assays of anxiety (Beckers et al., 2013; Ennaceur, 2014; Ennaceur & Chazot, 2016; Fonio et al., 2012). This is in part, because multiple affective states and motivations can result in the same observed behavior. For example, it is unclear whether increased locomotor activity in the center of an open field maze is due to a subjects’ low level of trait anxiety, an internal drive to explore, or even motivation to escape the arena. Similarly, freezing at the perimeter of the open field arena could be caused by fear of the brightly lit, open space, an innate desire to avoid potential aerial predators, or unseen external factors.

Distinct motivations to freeze are evident across assays; freezing on one task will not necessarily predict freezing on another. For example, freezing during context conditioning does not predict freezing on an elevated plus maze (Ahn et al., 2013; Hilton et al., 2023). Thus, the same mechanisms that lead to freezing during fear conditioning do not necessarily contribute to freezing on more exploratory-based behavioral assays, supporting the observation that multiple neural circuits can trigger this behavioral state (Zelikowsky et al., 2018).

More generally, the observable behaviors on these assays often are influenced by a variety of “hidden” environmental factors (Butler-Struben et al., 2022; Vogt et al., 2022) as well as peripheral signals from outside the brain, including peripheral organs, gut microbiota, and immune systems (Haroon et al., 2012; Koren et al., 2021; Kwon et al., 2021; Needham et al., 2022; Signoret-Genest et al., 2023; Tseng et al., 2023). All of these factors likely interact with the internal state of the animal to influence behavior, emphasizing that behavior does not have a one-to-one correspondence with affective state. As such, we advise caution when broadly interpreting findings from singular behavioral measures.

### The most adaptive behavior in a given situation can change depending on the context

Animals engage in behaviors that are determined to be the most optimal or adaptive strategy within the constraints of a specific task. For example, looming predators elicit freezing, presumably to avoid detection, whereas sweeping predators elicit escape, presumably because they believe they have been detected (De Franceschi et al., 2016; Lima & Dill, 1990). Even within the same assay, the defensive strategies used by subjects can evolve. For example, adaptive defensive strategies change depending on the proximity or imminence of the threat (Blanchard et al., 2011; Mobbs et al., 2020; Moscarello & Penzo, 2022). During the “pre-encounter” phase when potential predators loom, risk-assessment behaviors (e.g., rearing, exploration) are deemed adaptive. As the predator approaches and becomes more imminent, adaptive responses shift toward minimizing detection, including freezing during the “post-encounter” phase and, if necessary, attempting escape during the “circa-strike” phase (Blanchard et al., 2011). Consequently, the interpretation of “anxiety-like” behaviors can vary significantly depending on the specific context of each behavioral experiment.

There often is no singular advantageous, adaptive behavior in response to threat, with adaptive reactions dependent upon an ever changing environment (Holley & Fox, 2022; Holmes & Patrick, 2018). This adaptability is a key aspect of threat regulation and one that is often dysregulated in patients with anxiety (Moscarello & Maren, 2018). In short, behavioral output represents a complex cost/benefit analysis and the most adaptive behavior in a situation is uniquely individual at a given moment (Holley & Fox, 2022). These considerations are important to ensure that animal studies are most relevant to human anxiety.

### On the importance of multiple measures and contexts

In short, there is no one measure or assay that fully captures the experience of fear and anxiety in humans or animals. The concerns outlined above apply to freezing in rodents, just as well as they do to reaction time and/or amygdala BOLD activation in humans. This does not undermine the utility of individual assays. Rather, it serves as a cautionary tale about the overinterpretation of individual assays in restricted laboratory settings. Anxiety disorders are heterogeneous and persist across varied contexts, and translational research should take an equally heterogeneous approach. By incorporating various species, assays, and measures, translational research can be more than the sum of the individual measures and make great progress toward elucidating the neurobiology that contributes to anxiety disorders. When interpreting results from individual contexts or which report individual measures, it is important that we understand that this is simply a part of the puzzle, and simply “call a freeze a freeze.”
